# Naming a phantom – the quest to find the identity of *Ulluchu*, an unidentified ceremonial plant of the Moche culture in Northern Peru

**DOI:** 10.1186/1746-4269-5-8

**Published:** 2009-03-31

**Authors:** Rainer W Bussmann, Douglas Sharon

**Affiliations:** 1 William L Brown Center, Missouri Botanical Garden, PO Box 299, St. Louis, MO 63166-0299, USA; 2 2328 Dolphin Dr, Richmond, CA 94804, USA

## Abstract

The botanical identification of *Ulluchu*, an iconic fruit frequently depicted in the art of the pre-Columbian Moche culture that flourished from A.D. 100–800 on the Peruvian north coast, has eluded scientists since its documentation in ceramics in the 1930s. Moche fine-line drawings of *Ulluchu *normally depict seed-pods or seeds floating in the air in sacrificial scenes, associated with runners and messengers or intoxicated priests. It is a grooved, comma-shaped fruit with an enlarged calyx found mainly in fine-line scenes painted on Moche ceramics. The term first appeared without linguistic explanation in the work of pioneer Moche scholar Rafael Larco Hoyle, and the identification of the plant was seen as the largest remaining challenge in current archaebotany at the Peruvian North coast. The name *Ulluchu *seems to have been coined by Larco. According to his description, the name originated in the Virú River valley, and is supposedly of Mochica origin. However, there is no linguistic evidence that such a term indeed existed in the Mochica or Yunga language.

We conclude that *Ulluchu *can be identified as a group of species of the genus *Guarea *(Meliaceae) based on morphological characteristics. In addition, the chemical composition of the plant's compounds supports the thesis that it was used in a sacrificial context to improve the extraction of blood from sacrificial victims. We also suggest that a ground preparation of *Guarea *seeds, when inhaled, may have been used as a hallucinogen. However, more detailed phytochemical research is needed to corroborate the latter hypothesis.

## Background

*Ulluchu *is the common name assigned to a plant frequently depicted in the art of the Moche culture, which thrived on the north coast of Peru from A.D. 100 to 800. It is a grooved, comma-shaped fruit with an enlarged calyx found mainly in fine-line scenes painted on Moche ceramics. The term first appeared without linguistic explanation in the work of pioneer Moche scholar Rafael Larco Hoyle ([1] Fig. Fifty-eight: [2] Fig. Ninety-eight, Figs. One hundred and sixty-six and sixty-seven). In his 1939 publication, he reported that the peoples of the sierras and the coastal region (Viru and Moche valleys) believed that the fruit had to be picked silently to prevent it from turning bitter. He wondered if the plant symbolized the silence and discretion of richly attired Moche messengers, some of whom wear belts adorned with *Ulluchus*. In his 1938 publication, he labeled a Moche fine-line drawing of *Ulluchu *as *Phaseolus *sp. (a bean). Larco clearly recognized that *Ulluchu *had nothing whatsoever in common with "*ulluco*" (*Ullucus tuberosus*), an Andean tuber still widely cultivated and consumed in Peru nowadays.

The symbolic importance of *Ulluchu *in Moche iconography was firmly established by Moche scholar Donnan McClelland [3]. Based upon a meticulous review of the UCLA Moche Archive, she showed that its distribution was non-random and that its varied usage displayed definite patterns with the greatest variability among background elements and the most frequent representation found on the belts of warriors and runners. She demonstrated that "the leaves of the *Phaseolus *do not resemble the *ulluchu *leaf depictions" [[3]: 43]. *Pepino (Solanum muricatum) *and *aji (Capsicum annum)*, which are clearly depicted in Moche art and do not resemble *Ulluchu *were also eliminated "since the *ulluchi *[*sic*] fruit is suspended from the plant by its smaller pointed end, whereas these two are suspended by the large end" [[3]: 437]. She also indicated that the plant had not been botanically identified, pointing out that, if it turned out to be a mythical plant, no identification would be possible.

A decade after McClelland's seminal article, S. Henry Wassen [4] of the Gothenburg Ethnographical Museum, eliminated *Persea americana *Miller var. *americana *(a wild relative of *avocado*) as a candidate, and concluded that *Ulluchu *was *Carica candicans *A. Gray (a species of wild *papaya*). He also co-authored an article describing the enzyme papain, which can be extracted from unripe papaya, for use as a blood anti-coagulant [5]. In the latter article, the authors proposed that papain was used in the Moche sacrifice ceremony to prevent the coagulation of blood drawn from sacrificed warriors for later consumption by priests.

In a paper presented at the Sibley Conference at the University of Texas at Austin, in November 2003, McClelland [6], in addition to updating her 1977 paper in the light of a vastly expanded Moche Archive and archaeological discoveries of real *Ulluchu*, refuted the papaya hypothesis. She also discussed the presence in the art of yellow oleander seeds (*Thevetia peruviana*) as legging rattles as well as *espingo *seeds (*Nectandra *sp.) which [7] had earlier suggested might have been added to corn beer for medicinal and psychotropic purposes. McClelland concluded that the largest remaining challenge was the identification of *Ulluchu*.

In the present paper we build on the work of Donna McClelland and the archaeological excavations at Sipán in the Lambayeque Valley [8,9] and at Dos Cabezas in the Jequetepeque Valley in the 1990s [6].

## Materials and methods

The primary focus of this project has been the ethnobotany of medicinal plants used in Northern Peru and Southern Ecuador, and the changes that have occurred since early colonial times. Fieldwork for the present study was conducted in Southern Ecuador from 1995–2000, and in Northern Peru from 2001–2008. In the course of the fieldwork vouchers of all useful plant species sold in the markets of the region were collected after establishing prior informed consent. The specimens were registered under the collection series "RBU/PL," "ISA," "GER," "JULS," "EHCHL," "VFCHL," "TRUBH," and "TRUVANERICA," depending on the year of fieldwork and collection location. Vouchers of all specimens were deposited at the Herbario Truxillensis (HUT, Universidad Nacional de Trujillo), and Herbario Antenor Orrego (HAO, Universidad Privada Antenor Orrego, Trujillo). In order to recognize Peru's rights under the Convention on Biological Diversity, most notably with regard to the conservation of genetic resources in the framework of a study treating medicinal plants, the identification of the plant material was conducted entirely in Peru. No plant material was exported in any form whatsoever. The nomenclature of plant families, genera, and species followed the *Catalogue of the Flowering Plants and Gymnosperms of Peru *[10] and the *Catalogue of Vascular Plants of Ecuador *[11]. The nomenclature was compared to the TROPICOS database. Species were identified using the available volumes of the *Flora of Peru *[12], as well as [13,14] and reference material in the herbaria HUT, HAO, QCA, LOJA, and QCNE.

In addition to documenting the associated use-knowledge, plant collectors, vendors and *curanderos *(local healers) were interviewed about any possible knowledge of *Ulluchu*. Also, colonial records about useful plants of the region, which include [15-21] were searched for possible information and iconography of the plant. Photographs of all archeological specimens found, including photographs of the interior fruit structure in broken specimens, were obtained and used for direct comparison to botanical vouchers and life specimens of plant candidates. Ultimately, the available online collections of the Chicago Field Museum, New York Botanical Garden, and Missouri Botanical Garden were checked for possible candidates, and specimens from the Missouri Botanical Garden Herbarium were used for final determination and imaging.

## Discussion and Results

### Issues surrounding the name *Ulluchu*

The name *Ulluchu *seems to have been coined by Larco [1]. According to his description, the name originated in the Virú River valley, and is supposedly of Mochica origin. However, there is no linguistic evidence that such a term indeed existed in the Mochica or Yunga language. The most comprehensive Mochica-Spanish dictionary available, compiled from the writings of Moche scholar E. Brüning [22], has no such term. In addition, the local population, as well as market vendors, plant collectors, and *curanderos *interviewed, had no knowledge of *Ulluchu *whatsoever, other than what they derived from Larco. Since this first publication the term has been copied by all subsequent authors [e.g. [3,4]], without any regard to its validity. It is unlikely that Brüning [22] would have missed the name when doing his research early in the 20^th ^century, if it indeed was still being used. Brüning lists quite a few Mochica plant names, some of which are still used for the same plants today, e.g. "*faik*" = *Acacia macracantha *(*faique, espino*), from "*fáçek, fáçke*" = spine.

The only language that has a somewhat similar word from which *Ulluchu *could be derived is Quechua: "*uchu*" translates to "chili, pepper," while "*ullu*" translates as "penis." The term "*ullu uchu*" is sometimes used as a name for *Columellia ovata *R. & P. (Columelliaceae), a small high-Andean plant, described as "a very thick tree; its wood is suitable for various purposes, and its leaves have febrifugal properties and are very bitter" [21]. However, this plant has no likeness whatsoever to the Moche *Ulluchu*. Thus we must conclude that the term *Ulluchu *was most likely coined by Larco [[2]: 98] based on a Quechua term for a species with somewhat similar fruits that has no relation to the species used by the Moche.

### Botanical identification

#### Why is *Ulluchu *not *Carica candicans?*

*Carica candicans *is a wild relative of papaya (*Carica papaya *L.). Although the fruits are not marketed, they are occasionally consumed by the local population, and some market vendors and healers interviewed did know the plant under its vernacular name, "*mito*." Larco [1,2] never mentions the plant in relation to *Ulluchu*. Assuming that he indeed encountered a plant with that name, it cannot have been *C. candicans*, because this species would have been named "*mito*."

McClelland [6] argues that *Ulluchu "*cannot be a papaya, which belongs to a group of plants called 'cauliflory' [i.e., stem flowery]. ... The flowers and fruit of a cauliflory grow on the trunk of the tree and not on the limbs. ... *Ulluchus *depicted in Moche art, however, hang from limbs. Papaya leaves do not resemble *ulluchu *leaves, which are triangular, ovoid, or boomerang shapes hanging from limbs. Each large palmate papaya leaf grows on a stem from the top of the tree.*" *However, further complicating this matter, it turns out that *C. candicans *happens to be one of the few papayas that are not cauliflorous, that have triangular leaves with entire margins, and the fruits do hang from branches. Thus, judging from the iconography alone, *C. candicans *actually could be *Ulluchu*.

Based on recent archaeological evidence however, [6,8,9,23], it has become clear that the actual fruits found in burials do not resemble *C. candicans*. In addition, the explanation that papain, might have been extracted by the Moche from unripe papaya for use as a blood anti-coagulant [5], albeit reasonable, does not make much sense from a phytochemical perspective. Cultivated papaya (*C. papaya*) is often depicted in Moche pottery, and the species contains large amounts of papain. Why would the Moche have resorted to a rare wild species, when they could have used a cultivar with the same properties that grew on their very doorstep? Also, *C. candicans *fruits are often 10–15 cm. long, and, while this would relate to the size of some of the *Ulluchus *in the iconography, it is vastly larger than many of the fruits depicted and much larger than the fruits found in burials. Finally, the anatomy of *C. candicans *simply does not correlate with the fruits encountered in burials.

#### What is *Ulluchu*, and what was it really used for?

Moche fine-line drawings of *Ulluchu *normally depict seed pods or seeds floating in the air in sacrificial scenes (e.g., [6] Fig. Three.14), associated with runners and messengers (e.g., [6]: Fig. Three.1) or intoxicated priests (e.g., [6]: Fig. Three.6). The *Ulluchu *fruits vary greatly in size, ranging from about 1–15 cm. They are normally comma-shaped, often with an "exaggerated round calyx" [[6]: 43] with lines on the body of the fruit (e.g., [6]: Fig. Three.4). Some illustrations show *Ulluchu *harvested by monkeys, and in such cases the fruit is mostly shown growing along the axes of the plant's leaves (e.g., [6]: Figs. Three.27 & Three.28).

Starting from this basis in 2002, we built on the work of Donna McClelland and the archaeological excavations at Sipán in the Lambayeque Valley [8,9,24] and at Dos Cabezas in the Jequetepeque Valley in the 1990s [6,23]. Botanically, all these depictions resemble capsules or drupe-like fruits. It became apparent that in a biodiversity hotspot like Peru, with a flora of more than 18.000 species, a large number of plant families have fruits that vaguely resemble Moche fineline drawings of *Ulluchu*, and many of these families contain more than one genus with similar fruits. Examples include: (Apocynaceae: *Ambelania*; Caricaceae: *Carica*; Celastraceae: *Maytenus*; Chrysobalanaceae: *Chrysobalanus*, *Hirtella*, *Licania*; Convolvulaceae: *Dicranostyles*; Fabaceae: *Aldina*, *Alexa*, *Andira*, *Dipteryx*, *Dussia*, *Ormosia*; Guttiferae: *Tovomita*; Hippocrateaceae: *Cheiloclinum*, *Salacia*; Icacinaceae: *Calatola*; Meliaceae: *Guarea*; Menispermaceae: *Abuta*, *Curarea*, *Elephantomene*, *Telitoxicum*; Myristicaceae: *Virola*; Olacaceae: *Cathedra*; Quiinaceae: *Lacunaria*; Sabiaceae: *Meliosma*; Sapindaceae: *Cupania*, *Paullinia*; and Sapotaceae: *Pouteria*. Some of these are still highly important in traditional societies. For example, *Ambelania *fruit is often consumed; *Ormosia *contains potent poisonous compounds, but is now mostly used in crafts; *Curarea *is one of the ingredients of "*curare*," the famous Amazonian arrow poison; *Virola *species are still used as potent snuffs in the Amazon; and *Paullinia *is the source of "*yopo*," an important stimulant. However, none of these carry the vernacular name *Ulluchu*.

**Figure 1:**
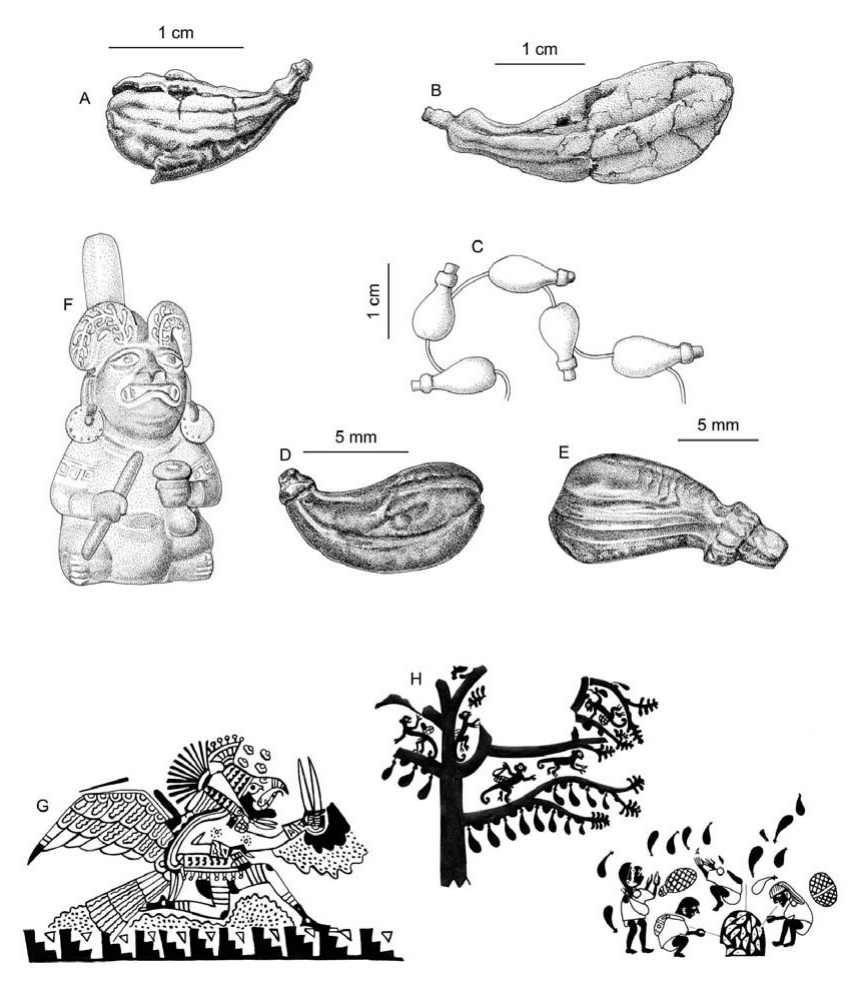
***Ulluchu *in archaeological context**. A. *Ulluchu *fruit from cache at Sipán. After photograph by Christopher B. Donnan. B. *Ulluchu *fruit from Dos Cabezas burial. After photograph by Donald McClelland. C. Bone beads in form of *Ulluchus *fron Huaca de la Luna. After photograph by Donald McClelland. D. Golden *Ullucho *bead. After photograph by Donald McClelland. E. *Spondylus *shell bead in form of *Ulluchu*. After photograph by Donald McClelland. F. Supernatural figure seated holding a gourd, possibly containing ground *Ulluchu *seeds with *Ulluchus *painted on headdress. Private collection. After photograph by Christopher B. Donnan, in McClelland ([6]: Fig. Three.18). G. Anthropomorphized hawk runner carrying a snuff tube with *Ulluchus *on its belt and *Ulluchu *seeds floating above it. The Art Institute of Chicago. After drawing by Donna McClelland in McClelland ([6] Fig. Three.12). H. *Ulluchu *harvest. Note tree with opposite leaves and extracted seeds on bottom right. After McClelland ([6]: Fig. Three.34), Private collection.

**Figure 2:**
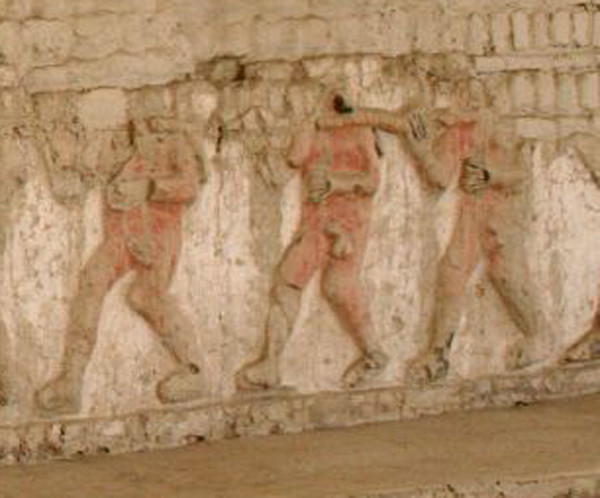
**Line of prisoners at Huaca El Brujo**. Photo by Rainer W. Bussmann.

Fortunately, at this time the archaeological evidence provides good clues for identification. The *Ulluchu *fruits found in burials in the 1990's are clearly capsules or drupes, slightly comma-shaped, between 1.5 and 5 cm. long, and slightly grooved (Fig. 1A&1B). They closely resemble bone, gold, and *Spondylus *beads found *in situ *in size, form, and texture (Fig. 1C–E). In the iconography, the fruits are often depicted on both sides of branches, as in the headdress of Fig. 1F. It is important to note that this figure has widely extended nostrils, as is often seen in people inhaling hallucinogenic snuffs, and is holding a gourd and pestle. McClelland ([6]: Fig. Three.18) interpreted this as lime gourd used for chewing coca. We suggest that this might also be a gourd used to grind the seeds of *Ulluchu *for inhalation. Further iconographic evidence supports this hypothesis. Runners and messengers associated with *Ulluchu *are often winged – they literally fly, i.e., the inhalation of *Ulluchu *gives them wings. An excellent example is the runner depicted in Fig. 1G: The personage has *Ulluchu *on his belt, *Ulluchu *seeds floating above its head, and an instrument in his hand that closely resembles a typical double snuff tube that would serve to inhale powdered hallucinogenic substances. Thus, it seems possible that one of the uses of *Ulluchu *may have been as a mind-altering snuff. Another reason for identifying the seeds in the iconography as hallucinogenic *Ulluchus *is underscored by Fig. 1H: This is part of a famous Moche scene (see [6]: Fig. Three.34) where monkeys are picking fruits from an *Ulluchu *tree. It is important to note that the tree depicted has opposite leaves and that seeds are extracted from the fruit, possibly for roasting in a typical oven on the bottom right. The roasted seeds could then be ground to powder and inhaled. The fruits themselves seem to be 5-valved. The function of *Ulluchu *as a hallucinogen is reinforced by other imagery (e.g., [6]: Fig. Three.6), where personages, surrounded by *Ulluchu *fruits lie on the ground in what clearly appears to be an intoxicated state. In addition, prisoners in sacrificial scenes (e.g., [25]: Figs. Two.2, Two.3, Two.12 & Two.24), especially the well known "lines of prisoners" at Huaca El Brujo and Huaca de la Luna (Fig. 2) all show clearly visible erections, which may be seen as another indication of the ingestion of some substance causing this effect. From this perspective Larco's term *Ulluchu *[2], if derived from Quechua "*ullu-uchu*" – "penis pepper," would in fact make sense in describing the possible effects of the plant in question. Also, the association with sexual arousal is reinforced by mythical scenes where an *Ulluchu *tree grows out of the back of an erotic couple.

**Figure 3:**
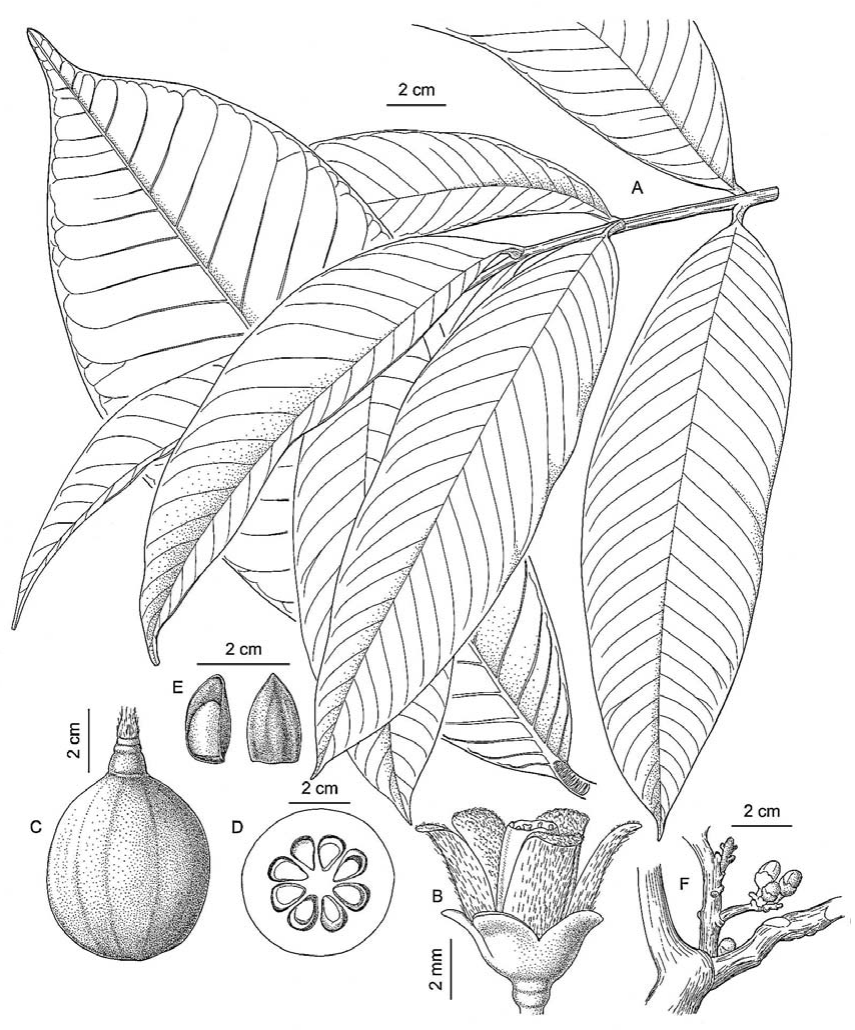
**Guarea grandifolia**. A. Mature branch. B. Flower. C. Mature fruit. D. Fruit cross section. E. Seeds F. Branching pattern.

**Figure 4:**
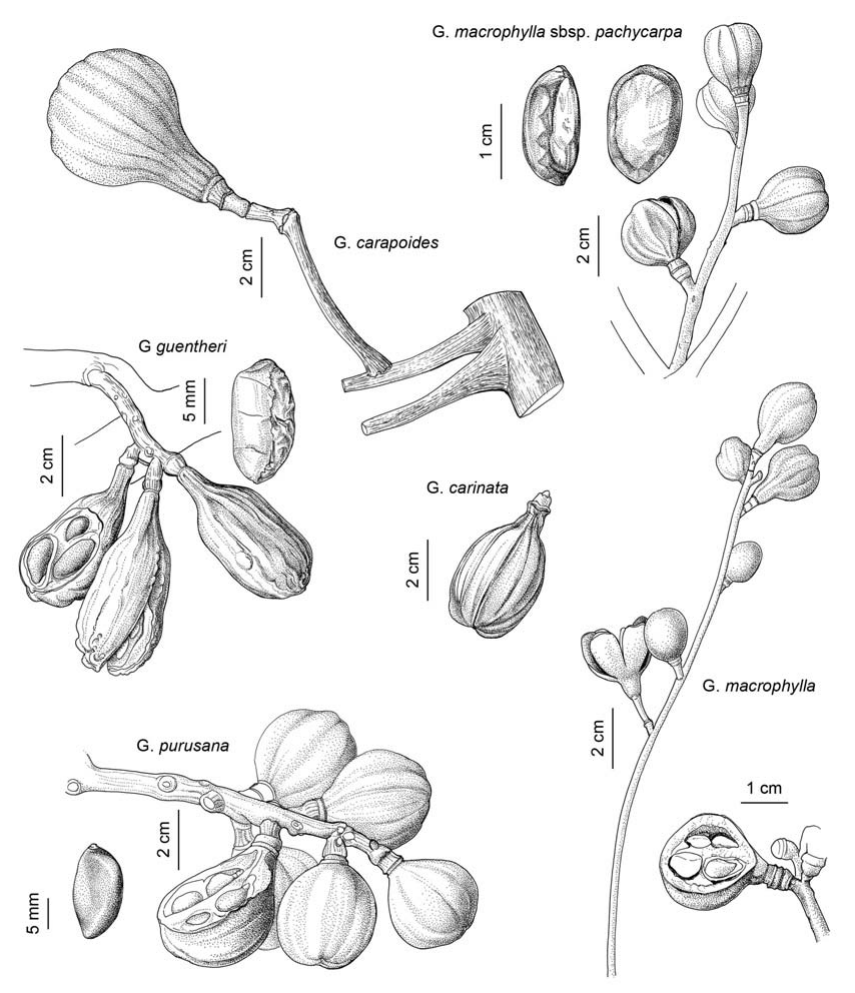
**Fruits of various species of *Guarea *(*G. macrophylla *with seeds, *G. carapoides*, *G. carinata*, *G. macrophylla*, *G. purusana *with seeds, *G. guentheri *with seeds)**.

In light of the above, *Ulluchu *is a tree with opposite leaves and fruits that are drupes between 1–15 cm long possibly containing active ingredients that would elevate the blood pressure and cause erections, and psycho-active substances. The only plant family from the list above having representatives that meet all these criteria is Meliaceae, and the genus *Guarea *is the one that most closely fits the description. It includes mostly trees with pinnate leaves (which is unusual for Meliaceae), and fruits that are 3–5 valved capsules, with large, pseudo arillate seeds. The genus *Guarea *is found throughout Peru, but is mostly restricted to tropical lowland forests, with some species reaching cloud forest habitat. No species is found along the dry coast of Peru, which indicates that the material must have been widely traded in Moche times. A typical representative is *Guarea grandifolia *DC. The species has clearly pinnate leaves (Fig. 3A), and the fruits (Fig. 3C) very clearly resemble the archaeological samples depicted in Fig. 1. In addition, *Guarea *contains a large number of species with varying fruit sizes (from 1–15 cm.), calyx swelling, and grooving on the body of the fruit (Fig. 4), which all correlates with the varied *Ulluchu *imagery in Moche fineline drawings. The seeds of *Guarea *species, with a distinct white navel, very much resemble the seeds depicted in Moche fineline paintings.

Many species of *Guarea *are known by a wide variety of vernacular names, e.g., *Guarea *spec.: requia, kushímsakish; *G. glabra*: yecheñor, yechemor; *G. grandifolia*: bola requia; *G. guidonia*: atapio, latapi, latapi caspi, requia colorada, requia latapi, xoro; *G. kunthiana*: requia, paujil ruro; *G. purusana*: latapi, requia). The wood of many species is used as timber for construction. The wood, bark, and leaves contain compounds that act as abortive, emetic, purgative, and antiamoebiac agents, and the bark is often sold as *Coccilliana *in expectorant preparations [12,26-28]. The fruits and seeds contain a variety of alkaloids that are very rarely used due to their high toxicity [29]. Some of the alkaloids found, e.g. rusbyine, have a structure and effects like emetine, an alkaloid found in *Psychotria ipecacuanha *(Brot.) Stokes, which has been widely used as an emetic and expectorant. Other species of *Psychotria *are well known as components in *ayahuasca *preparations due to their high content of N,N-DMT [30]. In large dosages, *ipecac *preparations cause high blood pressure, arrhythmia, spasms, and extension of the blood vessels. While the existing literature on *Guarea *seed compounds is rather fragmentary, it seems clear that a concentrated dosage of *Ulluchu *seeds, if ingested, would increase a prisoner's heartbeat, elevate the blood pressure, and widen blood vessels thus causing erection. All of this would make it much easier to extract sacrificial blood. Also, when inhaled by priests, the active compounds could have a mind-altering effect, which would not necessarily lead to high levels of toxicity, and could induce very rapid, short-term hallucinations.

## Conclusion

We conclude that *Ulluchu *can be identified as a group of species of the genus *Guarea *(Meliaceae) based on morphological characteristics. In addition, the chemical composition of the plant's compounds supports the theses of [3,4] that it was used in a sacrificial context to improve the extraction of blood from sacrificial victims. We also suggest that a ground preparation of *Guarea *seeds, when inhaled, may have been used as a hallucinogen. However, more detailed phytochemical research is needed to corroborate the latter hypothesis.

## Declaration of Competing interests

The authors declare that they have no competing interests.

## Authors' contributions

Both authors share the contributions to fieldwork, data analysis, and compilation of this manuscript. All authors read and approved the final manuscript.
